# Self-Assembling Polymers with *p*-Aminosalicylate Anions Supported by Encapsulation of *p*-Aminosalicylate for the Improvement of Drug Content and Release Efficiency

**DOI:** 10.3390/ph16101502

**Published:** 2023-10-23

**Authors:** Shadi Keihankhadiv, Dorota Neugebauer

**Affiliations:** Department of Physical Chemistry and Technology of Polymers, Faculty of Chemistry, Silesian University of Technology, 44-100 Gliwice, Poland; shadi.keihankhadiv@polsl.pl

**Keywords:** polymeric carrier, poly(ionic liquid), anti-tuberculosis, drug delivery system, choline, self-assembly

## Abstract

Bioactive linear choline-based copolymers were developed as micellar carriers for drug delivery systems (DDSs). The polymethacrylates containing trimethylammonium groups with *p*-aminosalicylate anions (PAS-based copolymers: series 1) or chloride anions (Cl-based copolymers: series 2) differing in ionic content and chain length were selected for drug loading. The diverse structures of amphiphilic copolymers made it possible to adjust the encapsulation efficiency of a well-known antibiotic, i.e., *p*-aminosalicylate in the form of sodium salt (PASNa) or acid (PASA), providing single drug systems. Goniometry was applied to verify the self-assembly capacity of the copolymers using the critical micelle concentration (CMC = 0.03–0.18 mg/mL) and the hydrophilicity level quantifying the surface wettability of polymer film using the water contact angle (WCA = 30–53°). Both parameters were regulated by the copolymer composition, indicating that the increase in ionic content caused higher CMC and lower WCA, but the latter was also modified to a less hydrophilic surface by drug encapsulation. The drug content (DC) in the PAS-based polymers was increased twice by encapsulation of PASNa and PASA (47–96% and 86–104%), whereas in the chloride-based polymer systems, the drug was loaded in 43–96% and 73–100%, respectively. Efficient drug release was detected for PASNa (80–100% series 1; 50–100% series 2) and PASA as complete in both series. The strategy of loading extra drug by encapsulation, which enhances the drug content in the copolymers containing anions of the same pharmaceutics, provided promising characteristics, which highlight the potential of PAS-loaded micellar copolymers for drug delivery.

## 1. Introduction

Polymers in the development of nanocarriers have garnered significant attention, and their potential in the field of medical applications is extensive, including in drug delivery systems (DDSs) [1,2]. The structures of polymers play a crucial role as they can be effectively utilized to engineer nanoparticles with a wide range of morphologies and architectures [3,4]. The unique feature of polymer carriers is attributed to their nanodimensions [5], which enables them to reach targeted cells and even intracellular organelles [6]. Drug-loaded polymer nanocarriers have gained approval for treating a wide range of diseases [7], mostly by the design of systems for anticancer therapy, tumor-targeted immunotherapy, and regenerative medicine or modern vaccines [8]. The polymer carriers can enhance the solubility of drugs, improving their efficient loading and intracellular absorption, and minimize undesired interactions between drugs and cells, concurrently exhibiting optimal stability, biocompatibility, and optional biodegradability. Drugs can be conjugated with polymers [9] and/or loaded in the polymer nanostructures, such as micelles [10], gels [11], vesicles [12], dendrimers [13], capsules [14], etc.

Polymeric micelles are common and stable nanostructures formed by amphiphilic macromolecules in aqueous environments [15,16]. Their self-assembling behavior allows for the formation of various types of polymeric micelles, depending on the composition of copolymers, the nature of interchain interactions, and the micellization method. The amphiphilic polymers demonstrate a distinct advantage in carrier design [17] in comparison to low molecular weight surfactants. Their lower values of critical micelle concentration (CMC) indicate increased stability and enhanced performance of polymeric micelles, ensuring efficient drug encapsulation, protection, and controlled release, which make them highly desirable in drug delivery applications.

The specific groups of amphiphilic copolymers are recognized as poly(ionic liquid)s or polymerized ionic liquids (PILs). They are obtained from ionic liquids (ILs), which are liquid salts that consist of organic cations and anions [18,19,20]. ILs are widely recognized for their exceptional properties, which include low vapor pressure at room temperature, diverse solubility profiles, non-flammability, high thermal stability, chemically inert behavior, adaptable polarity, variable viscosities, and other customizable characteristics [21,22,23,24,25]. In particular, choline, which is a trimethylammonium salt with a chloride anion and is water soluble, is often used as a naturally produced cationic component in biocompatible ILs with antibacterial properties [26,27,28,29]. Generally, choline-based ILs have the potential to improve the pharmacodynamic and pharmacokinetic properties of the carried drug [30]. A commercial choline ester derivative, that is [2-(methacryloyloxy)ethyl] trimethylammonium chloride (ChMACl) as a choline-based monomeric IL (MIL), exhibits considerable potential in the synthesis of PILs [31]. It has been employed in controlled radical polymerization to achieve choline-based PILs as the universal matrices, which can be modified to pharmaceutically active polymeric systems by chloride anion exchange into the anions of drugs like fusidate [32,33], clavulanate [33,34], sulfacetamide [35], piperacillin [31], and *p*-aminosalicylate (PAS) [33,34]. The strategy of ion exchange has also been applied in the choline-based MIL to introduce a pharmaceutical anion. The polymerization of choline MILs modified with pharmaceutical anions, such as salicylate [31,36,37], fusidate [38], cloxacillin [38], and PAS [39], have been reported. Cytotoxicity tests on the choline-based copolymers have demonstrated their non-toxic effects on normal BEAS-2B and cytotoxic activity against lung cancer cell lines [40,41].

Extensive studies have been conducted on PIL micelles and their uses for the encapsulation and delivery of active pharmaceuticals, e.g., curcumin [42,43], paclitaxel [44], doxorubicin [45,46], dopamine [47], acyclovir [48], etc. The unique combination of amphiphilicity in IL-based copolymers facilitates the development of systems with dual pharmacological actions. In this particular situation, the presence of the ionic drug within the PIL conjugate grants it a certain level of biological activity. However, by encapsulat-ing a non-ionic second drug into the core of the micelle, the overall biological activity of the system can be significantly enhanced. The micellar polymer conjugates working as dual-drug delivery systems have been studied for choline-based PILs with fusidate anions and then encapsulated with rifampicin non-ionic drug, which has been released at pH 7.4 during two days as 31–55% and 19–31%, respectively [32]. Similarly, the salicylate anions and encapsulated erythromycin have been combined in the polymer matrix, exhibiting a drug release of 40–50% and 60–70%, respectively, within a three-day period [35]. These polymer systems showed potential for combination therapy, particularly against drug-resistant strains, offering the advantage of delivering two drugs simultaneously in a single formulation, which eliminates the need for separate drug delivery.

**Figure 1:**
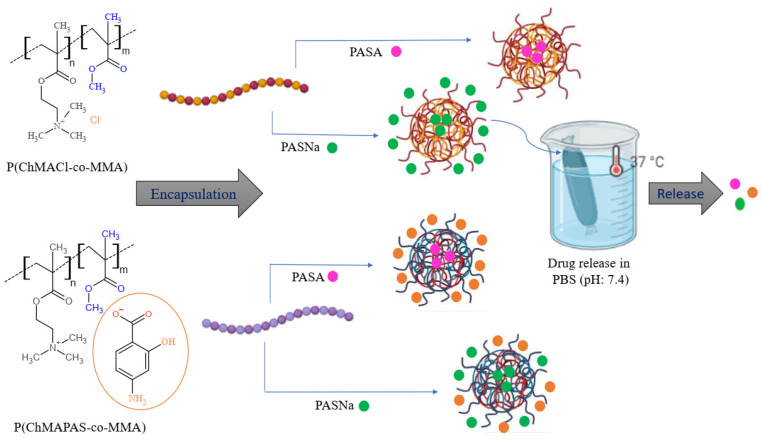
Schematic route to drug delivery micellar systems via encapsulation of PAS in the form of sodium salt (PASNa) and acid (PASA) by linear copolymers based on ChMACl and ChMAPAS.

In the present study, we investigate the use of amphiphilic linear choline-based copolymers as a matrix for the encapsulation of drugs to form micellar drug-loaded systems (Figure 1). In our previous work, we have reported the synthesis of these copolymers utilizing polymerizable MILs, ChMACl and [2-(methacryloyloxy)ethyl]trimethylammonium *p*-aminosalicylate (ChMAPAS) [39]. The amphiphilic nature of the matrix created an advantageous environment for the encapsulation of the selected drug, that is PAS in the form of acid (PASA) and sodium salt (PASNa) with antibacterial properties [49,50,51]. Motivated by the favorable characteristics of the choline-based copolymers, we embarked on exploring their potential as loaded micelles for the delivery of PAS, aiming to enhance their therapeutic effectiveness in the copolymer systems. In the case of PAS-based copolymer conjugates (series 1) already containing the drug, they were enriched extra by encapsulation with PAS, whereas in the chloride-based copolymers (series 2), the pharmaceutical activity was generated by PAS encapsulation. Our investigations involved a comparison of the micellar performance of DDSs containing PASA and PASNa, evaluating their efficacy in drug delivery. In order to assess the potential enhancement in therapeutic efficacy, we conducted in vitro drug release studies from the micelles in phosphate buffered saline (PBS) under conditions designed to simulate human body fluids at pH 7.4.

## 2. Results and Discussion

### 2.1. Encapsulation and Self-Assembly-Based Polymer Matrix

**Figure 2:**
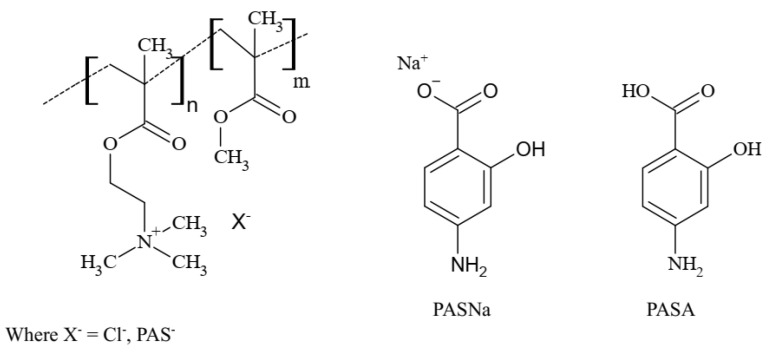
Structures of polymer matrices and encapsulated drugs.

The amphiphilic linear choline-based copolymers (Table 1, Figure 2) with PAS anions (series 1) and Cl anions (series 2) were used as matrices for encapsulating drugs, resulting in the formation of micellar drug-loaded systems (Figure 1). Previously, these copolymers have been designed by utilizing polymerizable ILs, ChMACl and ChMAPAS, by its copolymerization with methyl methacrylate (MMA) via atom transfer radical polymerization (ATRP) [39]. The amphiphilic properties of the polymer matrix provided a favorable environment for drug loading. In this case, PAS (Figure 2), both in acid form (PASA) and sodium salt (PASNa), were selected for encapsulation studies to explore their potential by enhancement of the therapeutic efficacy in the polymer systems. Both PASA and PASNa can be physically trapped in the self-assembled polymers of ChMAPAS and ChMACl, but the presence of chloride anions in the latter series makes them additionally beneficial for ionic exchange with the sodium salt of the drug, providing ionic conjugates (Figure 1). Because of that, we focused on three types of drug-loaded systems: (i) PAS-based copolymer conjugates with the ionically incorporated drug in the polymer (drug-loaded) and extra loaded PAS by encapsulation, (ii) chloride-based copolymers (non-loaded drug) with PASA physically introduced by encapsulation, and (iii) chloride-based copolymers (non-loaded drug) with PASNa physically and ionically introduced by encapsulation. The analysis of CMC and WCA techniques, as shown in Table 2, played a crucial role in this approach. By evaluating and comparing the performance of the drug-loaded micelles, valuable insights into their potential for delivering PAS and improving the overall efficiency of the drug delivery system were investigated.

### 2.2. Amphiphilic Properties and Wettability

**Figure 3:**
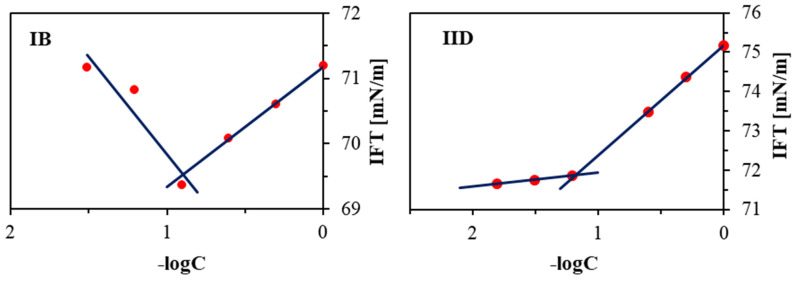
The variation of surface tension with the logarithm of the concentration of linear copolymer IB and IID in an aqueous solution.

The critical micelle concentration (CMC) was employed to evaluate the ability of the linear copolymers (IA-D and IIA-E) to form self-assembling micellar structures in aqueous solution, which confirms their amphiphilic nature and stability. The measurements of the interfacial tension (IFT) using the pendant drop method on a goniometer were performed for the series solutions with copolymer concentrations in the range of C = 0.03–0.18 mg/mL. The crossover point on the plot of IFT vs. logC (Figure 3) was used to determine the CMC value as the concentration at which the copolymer starts to self-assemble and form micelles, indicating amphiphilic behavior.

**Figure 4:**
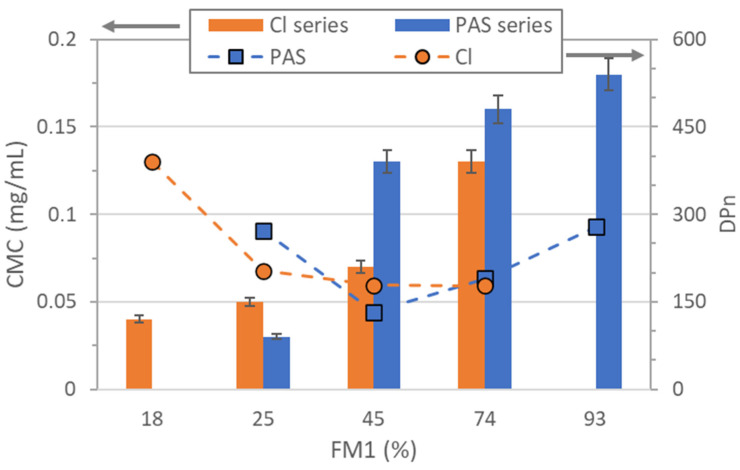
Dependence of ionic fraction content in the copolymers (F_M1_) on CMC in relation to the chain length (DP_n_) for both series of linear copolymers.

The CMC results shown in Figure 4 indicate the influence of copolymer structures, including the ionic content, on the amphiphilic behavior of the system. The chloride-based copolymers containing 18–74% of the ionic fraction exhibited CMC values ranging from 0.04 to 0.13 mg/mL, while the CMC for PAS-based copolymers with the higher maximum of ionic content (25–93%) was correlated to a broader range of concentration (0.03 to 0.18 mg/mL).

Additionally, for both series of linear copolymers, there was a noticeable trend indicating that the CMC value increased with the increase in the ionic fraction content. The nature of anions in the copolymer matrix is another significant factor, which could affect the interactions between the copolymer chains and alter the overall self-assembly behavior. Comparing the analogical co-polymers with similar content of PAS vs. chloride anions, the latter ones were expected to be slightly more hydrophilic and better soluble in water. Such a relation was observed for a pair of IA and IIB copolymers (F_M1_ ~ 25%), where the presence of PAS anions yielded less hydrophilic polymer and its self-assembling in lower concentration (0.03 vs. 0.05 mg/mL, respectively). Even though IB vs. IID and IC vs. IIE with similar ionic fraction contents (~45% and 74%, respectively) displayed the opposite CMC correlation (0.13 vs. 0.07 mg/mL and 0.16 vs. 0.13 mg/mL, respectively), suggesting the influence of the relative chain lengths, the chloride-based polymers in both pairs were characterized by higher or comparable DP_n_ below 200 of repeating units (133 vs. 179 and 190 vs. 178) in contrast to the pair of IA vs. IIB (272 vs. 203). Overall, the copolymers exhibited low CMC values, which are favorable for self-assembly behavior, making them promising candidates for the encapsulation of drugs in the micellar ionic polymers.

**Figure 5:**
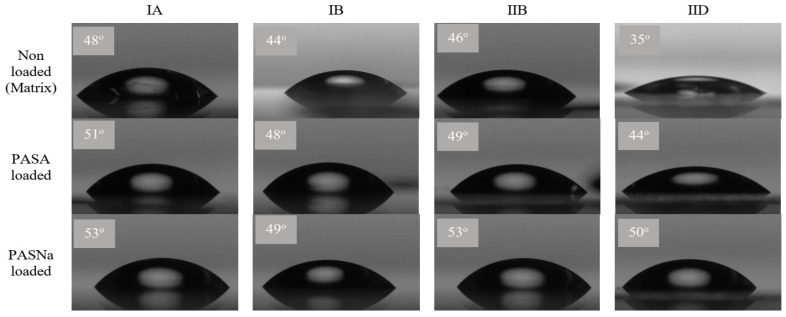
Snapshots of measuring WCA of a sessile drop using goniometer for various systems of IA-B and IIB-D.

**Figure 6:**
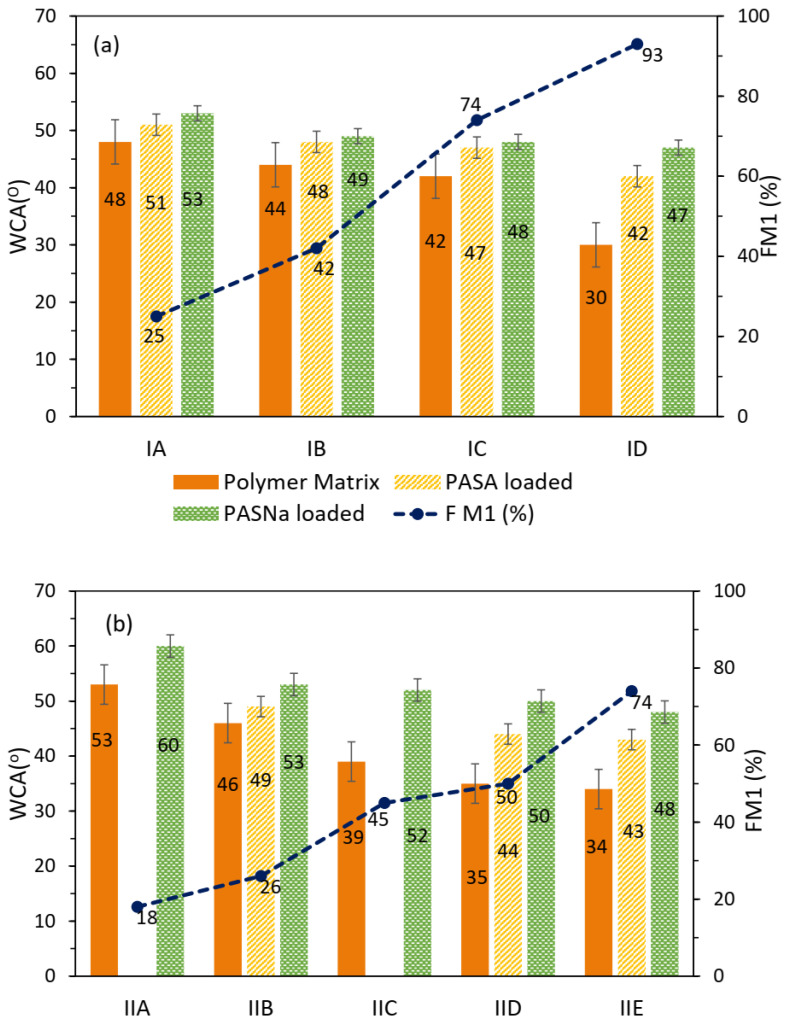
Dependence of WCA in relation to FM1 for both types of polymer matrices, ChMAPAS-based IA-D (**a**) and ChMACl-based IIA-E (**b**), in the systems without encapsulated drug and encapsulated with PASA or PASNa.

Hydrophilicity degree defining the dissolution ability of the amphiphilic copolymers in water was assessed by the wettability of their film surfaces, where a water droplet was placed to measure WCA (Table 2). The wettability changes are visually demonstrated in the photos for representative polymer samples with different types of anions and content of ionic fraction (Figure 5). The increase in ionic fraction content resulted in the decrease in WCA values for PAS polymer series 1 from 44° to 30° and for chloride polymer series 2 from 53° to 44°. Additionally, the PAS anions in comparison to Cl ones provided reduced interaction of water with the polymer surface as less wettable, exhibiting higher contact angles as was demonstrated by IA vs. IIB, IB vs. IIC-IID, and IC vs. IIE (Figure 6). The hydrophilic character of the self-assembling copolymers can also be changed by encapsulation of the drug. In our studies, the used PASNa is less hydrophilic than PASA, which was confirmed by higher WCA for the systems with encapsulated PASNa, whereas the PASA-encapsulated systems showed higher WCA than those of the non-encapsulated polymer matrices. It means that the WCA values were ordered as the following: non encapsulated < PASA encapsulated < PASNa encapsulated, both for the PAS- and chloride-based systems with comparable ionic content. The most spectacular differences in WCA were observed for the systems based on ID, that is 30° vs. 42° vs. 47°, respectively. These findings also suggest different molecular arrangements of polymer chains and surface characteristics.

### 2.3. Drug Content in Micellar Copolymers

**Figure 7:**
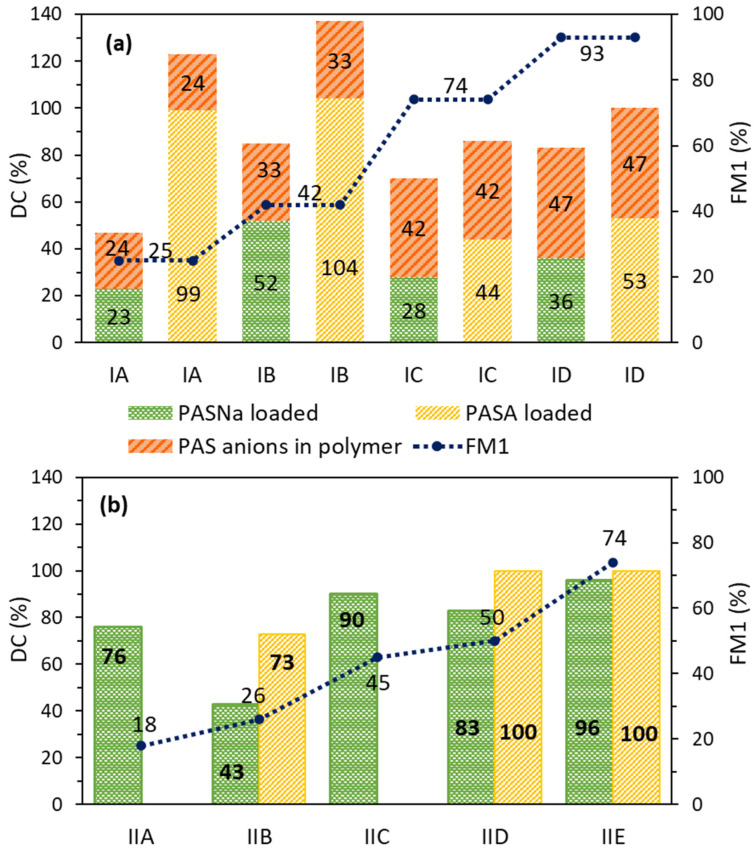
Dependence of drug content (DC) in the polymeric systems in relation to the content of ionic fraction (F_M1_) in PAS-based copolymers IA-D (**a**) and chloride-based copolymers IIA-E (**b**).

The UV–vis measured drug content (DC), which refers to the amount of introduced drug in copolymer micelles, can assess the efficiency of drug loading. The total DC varied depending on the chemical nature of the pharmaceutical substances and differences in the polymer composition, including the anion type. The values for both series were remarkable, achieving 47–86% (series 1) and 43–96% (series 2) for PASNa-loaded systems, as well as 86–137% (series 1) and 73–100% (series 2) for PASA-loaded systems (Table 2, Figure 7).

However, the micellar systems based on IA-D are the unique combination of different drug binding due to the PAS counterions introduced via polymerization of ChMAPAS monomer into the trimethylammonium polymethacrylate matrix (24–47% of ionically loaded PAS anions [39]) and that then encapsulated PASNa (23–52%) or PASA (44–104%) through physical interactions, which successfully contributed to improving the drug content efficiency of these systems. It suggests that the ionic conjugates of polymer–drug have the potential to accommodate a higher drug load. In the case of polymers in series 2, there is the possibility that during the encapsulation process, the sodium salt of PAS can also participate in the ionic exchange of chloride anions contained in the polymer, resulting in two types of drug binding, physically and ionically. Comparing the encapsulation ability by PAS-based vs. chloride-based polymers, the latter ones showed better efficiency, probably because of lower steric hindrance. Additionally, DC values are higher for the encapsulation of more hydrophilic PASA than for PASNa. This phenomenon can be attributed to the ability of the micelle to encapsulate an excess amount of drug beyond its theoretical capacity. The reduced steric hindrance allows for more efficient incorporation of PASA into the polymer matrix, leading to a higher DC.

### 2.4. Drug Release

**Figure 8:**
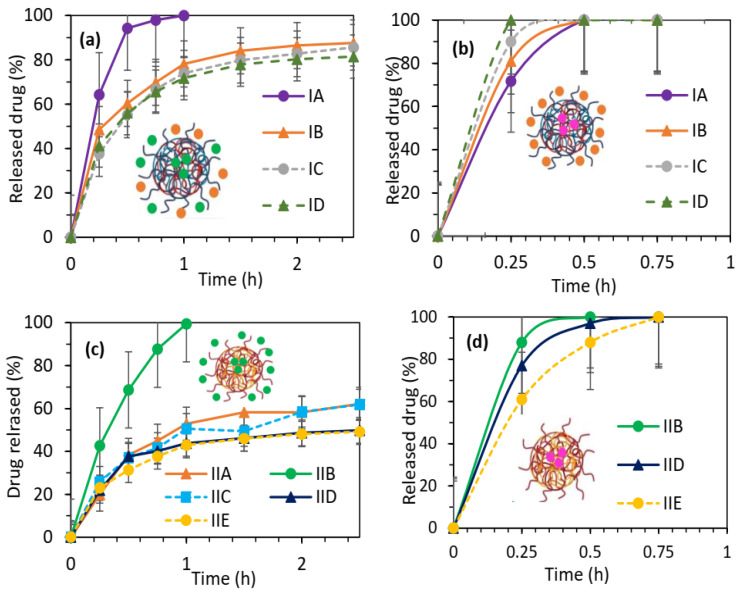
Drug release profiles for micellar systems of amphiphilic copolymers P(ChMAPAS-*co*-MMA)s (series 1) with encapsulated PASNa (**a**) and PASA (**b**), as well as P(ChMACl-*co*-MMA)s (series 2) with encapsulated PASNa (**c**) and PASA (**d**).

The in vitro drug release studies were conducted under physiological conditions (pH 7.4 at 37 °C) over a period of 72 h. The release of the drug from the samples was monitored at specific time intervals. The drug concentration in the release medium was determined using UV–vis spectrophotometry at λ = 265 nm. For the micellar systems based on PAS copolymers series 1 (Figure 8a) and chloride-based co-polymers series 2 (Figure 8c) with encapsulated PASNa, an initial burst release was observed within the first hour followed by a slower release over a period of up to 12 h. In most cases, the kinetic profiles reached a plateau after 1.5 h, demonstrating a controlled and sustained drug release from the micelles, whereas for IA and IIB, characterized by a low content of ionic fraction in the polymer matrices and the lowest DC of PASNa, complete drug release was attained within the first hour, indicating a rapid release from these systems. Comparing both series, it became evident that the amounts of released PASNa were higher for series 1 (80–100%) than those for series 2 (40–100%). In the case of systems with the encapsulated PASA, where the drug was released within half an hour in a percentage of 100% from series 1 (Figure 8b) and 88–100% from series 2 (Figure 8d), the polymer composition was influenced very slightly on the kinetic drug release.

It is worth to notice that in the PAS-encapsulated micelles formed by the conjugates of polymer containing PAS anions, the driving forces for drug release are attributed to the ionic exchange of PAS anions from the polymer conjugates by phosphate anions present in the PBS solution as well as to the diffusion process of the physically loaded drug by encapsulation within the polymer matrix. Previously investigated copolymers with PAS anions in a maximal content of 47% demonstrated 80% of drug release from IA, 98% from IB within 4 h, and complete release from IC-ID within 1 h [39]. Comparison of the release behavior of encapsulated vs. non-encapsulated systems indicates that the encapsulated drug slightly increases the total rate of released PAS.

The present results confirmed that the polymer matrix containing PAS anions with the extra loading PASNa or PASA enhanced the drug content in the micellar systems, which was correlated to a higher concentration of the released drug, providing a more efficient drug release process.

## 3. Materials and Methods

Methyl methacrylate (MMA), obtained from Alfa Aesar (Warsaw, Poland), was dried using molecular sieves and purged under argon gas. [2-(Methacryloyloxy)ethyl]trimethylammonium chloride (ChMACl), 80% aq. solution, purchased from Sigma-Aldrich (Poznan, Poland), was dried under reduced pressure until a constant weight was achieved. [2-(Methacryloyloxy)ethyl]trimethylammonium *p*-aminosalicylate (ChMAPAS) was prepared by anion exchange reaction, as has been described previously [39]. Ethyl 2-bromoisobutyrate (EBiB), *N,N,N′,N″,N″*-pentamethyldiethylenetriamine (PMDETA), and phosphate buffered saline (PBS) were obtained from Sigma-Aldrich (Poznan, Poland) and used as received. Copper (I) bromide (CuBr), 98%, from Fluka (Steinheim, Germany), was purified by stirring with glacial acetic acid, followed by filtration, washing with ethanol and diethyl ether, and drying under vacuum. Methanol (MeOH) from Chempur (Piekary Slaskie, Poland) and tetrahydrofuran (THF) from Sigma-Aldrich (Poznan, Poland), were dried using molecular sieves and purged under with argon gas. *p*-Aminosalicylate acid (PASA) and sodium *p*-aminosalicylate (PASNa), both with a purity of 98%, were obtained from Alfa Aesar (Warsaw, Poland) and used without further purification. Deionized water was obtained using equipment of Hydrolab HLP Uv5 (Straszyn, Poland).

### 3.1. Synthesis of Linear ChMA-Based Copolymers

The copolymerization of ChMACl or ChMAPAS and MMA with various molar ratios of comonomers (25/75, 50/50, 75/50) using EBiB as a monofunctional initiator and CuBr/PMDETA catalytic system in MeOH and THF solvent at various ratios of monomer to initiator (M:I= 400:1, 600:1) were performed using the atom transfer radical polymerization (ATRP), according to previously reported procedure [39]. The obtained copolymers, P(ChMAPAS-*co*-MMA) as the series 1 and P(ChMACl-*co*-MMA) as the series 2, were characterized using ^1^HNMR to confirm their structures, including evaluation of the ionic fraction contents (F_M1_), degree of polymerization (DP_n_), and to calculate molecular weights (M_n NMR_), whereas SEC was applied to determine their molecular weights (M_n SEC_) and dispersity indices (Đ_SEC_), as is presented in Table 1.

### 3.2. Polymer Micellization and Drug Encapsulation

The amphiphilic linear copolymer (20 mg) and PAS drug (PASA vs. PASNa, 20 mg) were dissolved in methanol (2 mL). Deionized water (4 mL, two-fold excess of water relative to the solvent) was added dropwise to the mixture, which was then stirred for 24 h. Afterward, the methanol was evaporated, and the resulting aqueous fraction was collected and next lyophilized by freezing to obtain a solid product.

### 3.3. Drug Release from Micellar Copolymer Systems

The polymer micelles (1.0 mg) were dissolved in 1 mL of PBS solution with a pH of 7.4. To conduct the drug release study, a dialysis cellulose membrane bag (MWCO = 3.5 kDa) was filled with 1 mL of micelle solution and placed in a glass vial containing 44 mL of PBS. The solution was stirred during the 72-h dialysis period at 37 °C. During the dialysis process, the progress of drug release was monitored by measuring its concentration in the receiving PBS solution outside the dialysis bag. Samples (1 mL) containing the released drug were collected at various time points. UV–vis spectroscopy was employed to analyze the samples and determine the amount of released drug by measuring absorbance at λ = 265 nm for PASNa and PASA. After analysis, the samples were returned to the glass vial to maintain a constant volume of the PBS medium. The calculations of the drug concentration in the release medium were performed using the Lambert–Beer law and the linear range of the calibration curve for the drug solution in PBS. Each reported result represents an average of three parallel measurements.

### 3.4. Characterization

Proton nuclear magnetic resonance (^1^H NMR) spectra data were acquired using a UNITY/NOVA spectrometer (Varian, Mulgrave, Victoria, Australia) operating at a frequency of 300 MHz. Deuterated dimethyl sulfoxide-d6 served as the solvent, whereas tetramethylsilane was employed as an internal standard. Size exclusion chromatography (SEC) measurements were carried out using an Ultimate 3000 chromatography (Thermo Fisher Scientific, Waltham, MA, USA) equipped with a precolumn TSKgel Guardian SuperMP (HZ)-H (4.6 mm × 2 cm, particle size of 6 μm), two columns of TSKgel SuperMilipore HZ-H (4.6 mm × 15 cm, particle size 6 μm), and a differential refractometer RefractoMax 521 detector. The analysis was conducted at 40 °C in de-ionized water with a flow rate of 0.45 mL/min using poly(ethylene oxide)/poly(ethylene glycol) standards ranging from 982 to 227,000 g/mol. Ultraviolet visible light spectroscopy (UV–vis) was employed using a spectrometer model Evolution 300 from Thermo Fisher Scientific (Waltham, MA, USA) to determine the content of anionic drugs (DC) in conjugates and micelles as well as to quantify the amount of drug released during in vitro studies. The measurements were performed using quartz cuvettes as the sample containers. The polymer sample in PBS at a concentration of 0.05 mg/mL was transferred to a quartz cuvette and measured at a wavelength of 265 nm. A calibration curve was generated using drug concentrations ranging from 0.1 mg/mL to 0.006 mg/mL in PBS. The critical micelle concentration (CMC) was assessed using the pendant drop method on a goniometer (OCA 15EC, DataPhysics, Filderstadt, Germany) to measure the interfacial tension (IFT). To determine the CMC, a series of aqueous polymer solutions with concentrations ranging from 0.003 to 0.1 mg/mL were prepared. The water contact angle (WCA) measurements were conducted using the sessile drop method on the same goniometer apparatus as mentioned above. A polymer solution in methanol with a concentration of 0.3 mg/mL was spin-coated onto a thin glass plate. Subsequently, a 4 µL droplet of de-ionized water was carefully placed on the thin polymer layer, and the contact angle was measured. The data obtained were collected and analyzed using the SCA20_U software (Version 2, DataPhysics Instruments GmbH, Filderstadt, Germany).

## 4. Conclusions

The amphiphilic linear polymethacrylates containing trimethylammonium groups with various counterions (pharmaceutically active PAS anions vs. chloride anions) were investigated to create micellar drug conjugate systems with physically encapsulated PAS drug (PASA in acidic form vs. PASNa sodium salt form). The amphiphilic copolymers in aqueous solutions demonstrated low CMCs, which increased with the ionic content and confirmed the self-assembling behavior with the ability to encapsulate the drug. The studies on polymer film surfaces indicated low WCAs, which showed the increase in hydrophilicity with the ionic fraction content, but after drug encapsulation, the hydrophilicity of the film surfaces was reduced, yielding slightly higher WCAs, 47–60° PASNa loaded vs. 42–51° PASA loaded. The PAS encapsulation enriched the drug content in the PAS-based copolymers (from 24–47% to 47–86% (PASNa) vs. 86–37% (PASA)), whereas the systems of chloride-based copolymers were activated pharmaceutically containing 43–96% of PASNa vs. 73–100% of PASA. These results suggest that the relatively high drug content in all systems makes them beneficial for PAS delivery, but the PASA encapsulation in the PAS-based polymers with lower ionic content and in the chloride-based polymers at higher content of ionic fraction are the most efficient systems. For most systems, the complete release of PAS was detected within 0.5–1 h, but it was more efficient for PAS-based copolymers than the chloride ones. Generally, presented polymer conjugate-based micellar systems demonstrated a great ability to encapsulate and release the selected drugs at a satisfactory level, which is promising for the improvement of their therapeutic efficacy.

## Figures and Tables

**Table 1:** Basic characteristics of copolymer matrices P(ChMAPAS-*co*-MMA) (series 1) and P(ChMACl-*co*-MMA) (series 2) [39].

No	F_M1_(%)	DP_n_/DP_M1_	M_n NMR_ (g/mol)	M_n SEC_ (g/mol)	Đ_SEC_
IA	25	272/68	42,500	65,900	1.29
IB	42	133/56	25,800	67,300	1.25
IC	74	190/139	50,300	51,600	1.33
ID	93	279/261	86,500	212,200	1.55
IIA	18	390/71	46,700	47,900	1.12
IIB	26	203/52	26,900	22,400	1.26
IIC	45	497/224	73,800	67,700	1.96
IID	50	179/90	27,600	29,600	1.13
IIE	74	178/132	31,900	29,900	1.14

IA-ID: M1 = ChMAPAS, IIA-IID: M1 = ChMACl; M2 = MMA; conditions: [M1+M2]_0_:[EBiB]_0_:[CuBr]_0_:[PMDETA]_0_ = 400:1:1:1 (except IC, ID, IIA, and IIC where [M1+M2]_0_:[EBiB]_0_ = 600:1), MeOH:ChMA = 1:1 (*v/w*), MeOH:THF=3:1 (*v/v*), 40 °C; F_M1_—content of ionic fraction in the copolymer; DP_n_/DP_M1_—total polymerization degree and polymerization degree of ionic units, respectively.

**Table 2:** Characteristics of copolymers by micellization ability and surface wettability properties as well as drug encapsulation and release data.

No	CMC (mg/mL)	WCA (°)			DC (%)			Released Drug (%)	
		Polymer Matrix	PASA Loaded	PASNa Loaded	PAS Anions in Polymer	PASA Loaded	PASNa Loaded	PASA	PASNa
IA	0.03	48	51	53	24	99	23	100	100
IB	0.13	44	48	49	33	104	52	100	85.6
IC	0.16	42	47	48	42	44	28	100	87.6
ID	0.18	30	42	47	47	53	36	100	81.5
IIA	0.04	53	-	60	0	-	76	-	99.5
IIB	0.05	46	49	53	0	73	43	100	61.9
IIC	0.06	39	-	52	0	-	90	-	49.2
IID	0.07	35	44	50	0	100	83	100	49.2
IIE	0.13	34	43	48	0	100	96	100	61.9

## Data Availability

Not applicable.

## Abbreviations/Nomenclature

ATRP	Atom transfer radical polymerization
DDS	Drug delivery system
WCA	Water contact angel
CMC	Critical micelle concentration
DC	Drug content
MMA	Methyl methacrylate
ChMACl	[2-(methacryloyloxy)ethyl]trimethylammonium chloride
ChMAPAS	[2-(methacryloyloxy)ethyl]trimethylammonium *p*-aminosalicylate
PASA	*p*-aminosalicylate acid
PASNa	*p*-aminosalicylate sodium salt
PBS	Phosphate buffered saline
IL	Ionic liquid
MIL	Monomeric ionic liquid
PIL	Polymerized ionic liquid
IFT	Interfacial tension
EBiB	Ethyl 2-bromoisobutyrate
PMDETA	*N,N,N′,N′,N′*-pentamethyldiethylenetriamine
CuBr	Copper (I) bromide
THF	Tetrahydrofuran
MeOH	Methanol
UV-Vis	Ultraviolet visible light spectroscopy
DP_M1_	Polymerization degree of ionic units
DP_n_	Polymerization degree
F_M1_	Ionic fraction contents
SEC	Size exclusion chromatography
Đ	Dispersity index
M_n_	Average molecular weight of polymer
